# An Incremental High-Utility Mining Algorithm with Transaction Insertion

**DOI:** 10.1155/2015/161564

**Published:** 2015-02-25

**Authors:** Jerry Chun-Wei Lin, Wensheng Gan, Tzung-Pei Hong, Binbin Zhang

**Affiliations:** ^1^School of Computer Science and Technology, Harbin Institute of Technology Shenzhen Graduate School, HIT Campus, Shenzhen University Town, Xili, Shenzhen 518055, China; ^2^Department of Computer Science and Information Engineering, National University of Kaohsiung, Kaohsiung 811, Taiwan; ^3^Department of Computer Science and Engineering, National Sun Yat-sen University, Kaohsiung 804, Taiwan; ^4^Medical School, Shenzhen University, Shenzhen 518060, China

## Abstract

Association-rule mining is commonly used to discover useful and meaningful patterns from a very large database. It only considers the occurrence frequencies of items to reveal the relationships among itemsets. Traditional association-rule mining is, however, not suitable in real-world applications since the purchased items from a customer may have various factors, such as profit or quantity. High-utility mining was designed to solve the limitations of association-rule mining by considering both the quantity and profit measures. Most algorithms of high-utility mining are designed to handle the static database. Fewer researches handle the dynamic high-utility mining with transaction insertion, thus requiring the computations of database rescan and combination explosion of pattern-growth mechanism. In this paper, an efficient incremental algorithm with transaction insertion is designed to reduce computations without candidate generation based on the utility-list structures. The enumeration tree and the relationships between 2-itemsets are also adopted in the proposed algorithm to speed up the computations. Several experiments are conducted to show the performance of the proposed algorithm in terms of runtime, memory consumption, and number of generated patterns.

## 1. Introduction

Association-rule mining (ARM) [1–3] from a transactional database is a fundamental task for revealing the relationships among items. The Apriori [4] was the first algorithm to mine the association rules in a level-wise way. It uses generate-and-test mechanism to find the candidate itemsets and then derive the frequent itemsets based on the minimum support threshold. The association rules are then revealed from the discovered frequent itemsets based on minimum confidence threshold. The FP-growth algorithm [5] was the first algorithm to efficiently mine the frequent itemsets without candidate generation. It uses the FP-tree structure to compress the original database into a tree structure. An index Header_Table with a designed FP-growth mining algorithm is also proposed to find the corresponding paths of the items for deriving the frequent itemsets. Many algorithms have been, respectively, proposed to efficiently mine the association rules based on either the level-wise or pattern-growth mechanisms [2, 3]. Both the level-wise or pattern-growth approaches can only handle the static database in batch mode. When transactions are changed in the database, new information may arise and old ones may become invalid. The updated database is required to be processed to mine the updated information in batch mode, which is not suitable in practical applications.

To solve the above limitations of batch-mode algorithms [6, 7], Cheung et al. proposed the Fast-UPdated (FUP) algorithm [8] to maintain and update the discovered information with transaction insertion. It divides the discovered frequent itemsets from the original database and all itemsets in the inserted transactions into four cases. The procedures for four cases are, respectively, designed to maintain and update the discovered frequent itemsets. When the itemsets are small in the original database (support ratio is lower than minimum support threshold) but large in the new database (support ratio is larger than or equal to the minimum support threshold), the original database is required to be rescanned to find the actual occurrence frequencies of the small itemsets in the original database.

For ARM, it only reveals the binary relationships among items. The implicit factors such as profit or quantity are not concerned in ARM. A pattern with highly frequency may not be interested if it cannot bring highly profit for retailer. For example, a sale of diamonds may occur less frequently than that of clothing in a department store, but the former gives a much higher profit per unit sold than the latter. Only the occurrence frequency is insufficient to identify highly profitable items in traditional ARM.

High-utility mining (HUM) [9, 10] was thus proposed to partially solve the limitations of association-rule mining. It may be thought of as an extension of frequent-itemset mining by considering the sold quantities and profits of the items. The utility of an itemset can be measured in terms of quantity and profit, which can be defined by user preference. For example, someone may be interested in finding the itemsets with good profits and another may focus on the itemsets with low pollution while manufacturing. When the utility of an itemset is larger than or equal to the minimum utility count, an itemset is considered as a high-utility itemset (HUI). Several algorithms have been proposed to mine HUIs in a static database [11–14].

As previously mentioned in ARM, it is also an important issue to design an algorithm to efficiently maintain and update the HUIs when data or transactions are frequently changed in the original database. Some HUM algorithms have been proposed with transaction insertion [15–17]. The original database is still, however, required to be rescanned for maintaining and updating the HUIs in some cases. The problem of combination explosion based on level-wise approach is also a critical issue to be solved.

In this paper, a memory-based incremental approach for maintaining and updating the discovered HUIs is proposed with transaction insertion. The proposed algorithm inherits the HUI-Miner algorithm [18] to build the utility-list structures for mining HUIs in incremental mining. Since the utility-list structure is a condensed way to keep the related information for high-utility mining, all itemsets whether they are high transaction-weighted utilization itemsets (HTWUIs) or small in the original database should be kept. An estimated utility cooccurrence structure (EUCS) [19] is also applied in the proposed algorithm to speed up the performance of the proposed approach. Based on the designed algorithm, it outperforms the two-phase algorithm [12] and the state-of-the-art FHM algorithm [19] in batch mode and other previous algorithms for incremental mining [16, 17].

The remaining of this paper is organized as follows. Related works are reviewed in Section 2. The preliminaries and problem statement are described in Section 3. The proposed incremental algorithm with transaction insertion is given in Section 4. An illustrated example to explain the proposed algorithm step-by-step is described in Section 5. Experiments are provided in Section 6. Conclusion is finally given in Section 7.

## 2. Review of High-Utility Mining

Traditional ARM only concerns the binary values of the itemsets in a transactional database. The frequent itemsets only reveal the occurrence frequencies of the itemsets in the transactions, which is not suitable in real-world applications. Other factors such as price, quantity, or cost can also be used as the important measurements to analyze and predict purchased behaviors of the customers. Besides, highly profitable products with lower frequencies may not be discovered in traditional ARM. For example, in the basket analysis, jewels and diamonds are high profitable items but may not be frequent compared to food or drink products.

High-utility mining (HUM) [9, 10] is concerned as an extension of the frequent itemsets mining by considering both the quantities and profits of items to discover the valuable itemsets than the frequent ones. An itemset is concerned as a HUI if its utility value is larger than or equal to the minimum utility count. Chan et al. first proposed the top-*k* objective-directed data mining to mine the top-*k* closed utility patterns based on business objective [9]. Not only the frequent itemsets but also the HUIs can be thus discovered by the designed approach. Yao and Hamilton proposed the utility model to firstly consider both quantities and profits of the items to mine the HUIs [10]. Several mathematical properties of utility constraints and two pruning strategies are also designed to efficiently mine HUIs. Liu et al. proposed the two-phase model [12] to mine HUIs based on the developed transaction-weighted downward closure (TWDC) property. Based on two-phase model, the numerous candidates can be greatly reduced and the high-utility itemsets can be precisely obtained.

Many algorithms have been proposed to mine HUIs based on two-phase model. Lin et al. designed a high-utility pattern- (HUP-) tree algorithm [11] to compress the original database into a tree structure. A pattern-growth HUP-growth mining algorithm was also designed to mine HUIs. Tseng et al. then proposed the UP-tree structure with UP-growth and UP-growth^+^ mining algorithms to efficiently mine HUIs [13]. Since the pattern-growth approach requires computations to trace the tree nodes in the tree structure, Liu and Qu then proposed a HUI-Miner algorithm [18] to compress the database into the utility-list structures. Each entry in the utility-list structure stores transaction IDs (TIDs), the utility of itemset *X* in the transaction (*Iutility*), and the rest utilities of itemsets except *X* in the transaction (*Rutility*). Based on the HUI-Miner algorithm and the designed pruning strategy of the enumeration tree, the HUIs can be easily discovered. Fournier-Viger et al. then modified the HUI-Miner algorithm and designed an estimated utility cooccurrence structure (EUCS) to keep the relationships between 2-itemsets, thus speeding up the computations compared to the HUI-Miner algorithm [19].

Most algorithms process the static database to mine HUIs. In real-world applications, transactions are dynamically changed in the original database. Ahmed et al. proposed an IHUP algorithm with three tree structures for mining HUIs with transaction insertion [15]. The proposed tree-based algorithm can be used to avoid the generate-and-test mechanism for HUM. The IHUP-tree algorithm still requires to generate numerous HTWUIs based on the pattern-growth approach. Lin et al. proposed an incremental (FUP-HUI-INS) algorithm [17] for updating the discovered HUIs based on the FUP concept [8] and two-phase model [12] with transaction insertion. Two parts with four cases are then divided by the HTWUIs in the original databases and all itemsets in the inserted transactions. Each case is then processed by the designed procedure to maintain and update the discovered HUIs. Although the FUP-HUI-INS algorithm has good performance than the two-phase model, the original database is still required to be rescanned when an itemset is small in the original database but HTWUI in the inserted transactions. To solve the limitations of FUP-HUI-INS algorithm, Lin et al. then proposed an improved prelarge concept for mining high-utility itemsets with transaction insertion (PRE-HUI-INS) [16]. Based on the property of prelarge concept [20], prelarge transaction-weighted utilization itemsets (PTWUIs) are kept to avoid database rescan until the cumulative total utility of the inserted transactions achieves the safety bound. Since FUP-HUI-INS and PRE-HUI-INS algorithms are processed by two-phase model, an additional database rescan is still necessary to be performed to find the actually HUIs. Besides, it requires computations to find the HTWUIs based on the pattern-growth approach.

## 3. Preliminaries and Problem Statement

In this section, the preliminaries related to HUM are given below.

### 3.1. Notations

 
*D*: original quantitative database, *D* = {*T*_1_, *T*_2_,…, *T*_*n*_}, in which *n* is the transactions number and each transaction includes a subset of items with quantities;
 
*d*: set of new transactions, *d* = {*t*_1_, *t*_2_,…, *t*_*n*_}, in which each transaction includes a subset of items with quantities;
 
*U*: entire updated database, that is, *D* ∪ *d*;
 
*I*: set of *m* items, *I* = {*i*_1_, *i*_2_,…, *i*_*m*_}, each item *i*_*j*_ with a profit value *p*_*j*_;
 TID: each transaction *T*_*n*_ ∈ *D* has a unique transaction identification;
 
*u*_*jk*_: utility value of each item *i*_*j*_ in each transaction;
 
*tu*_*k*_: accumulated utility value of the items in each transaction;
 
*q*_*jk*_: quantity of item *i*_*j*_ in each transaction;
 
*σ*: predefined minimum high-utility threshold;
 TWU^*D*^(*i*_*j*_): transaction-weighted utility of an item *i*_*j*_ in the original database *D*.

### 3.2. Preliminaries and Problem Statement

Assume an example database consists of 10 transactions and 6 items, and each item in the transaction has its purchased quantity. A used example is shown in Table 1. The profit table for the items is shown in Table 2.

In this example, the minimum utility threshold is set at 35%. The definitions of HUM are given below.

**Definition 1 .**

An itemset *X* is a set of *k* distinct items {*i*_1_, *i*_2_,…, *i*_*k*_}, in which *k* is the length of an itemset. An itemset *X* is contained in a transaction *T*_*n*_ if *X*⊆*T*_*n*_.

For example, an item (*A*) is called a 1-itemset which contained in *T*_1_, and an itemset (*ABD*) is called 3-itemset in *T*_1_.

**Definition 2 .**

The utility of an item *i*_*j*_ in *T*_*q*_ is defined as *u*(*i*_*j*_, *T*_*q*_) = *q*(*i*_*j*_, *T*_*q*_) × *p*(*i*_*j*_), in which *q*(*i*_*j*_, *T*_*q*_) is the quantity of an item *i*_*j*_ in *T*_*q*_, and *p*(*i*_*j*_) is the profit value of an item *i*_*j*_.

For example, the utility of an item (*A*) in *T*_1_ is *u*(*A*, *T*_1_) = *q*(*A*, *T*_1_) × *p*(*A*)  ( = 3 × 3)  ( = 9).

**Definition 3 .**

The utility of an itemset *X* in transaction *T*_*q*_ is denoted by *u*(*X*, *T*_*q*_), which can be defined as *u*(*X*, *T*_*q*_) = ∑_*i*_*j*_∈*X*∧*X*⊆*T*_*q*__ *u*(*i*_*j*_, *T*_*q*_).

For example, *u*(*AD*, *T*_1_) = *u*(*A*, *T*_1_) + *u*(*D*, *T*_1_) = *q*(*A*, *T*_1_) × *p*(*A*) + *q*(*D*, *T*_1_) × *p*(*D*)  ( = 3 × 3 + 3 × 50)  ( = 159).

**Definition 4 .**

The utility of an itemset *X* in *D* is denoted by *u*(*X*), which can be defined as *u*(*X*) = ∑_*X*⊆*T*_*q*_∧*T*_*q*_∈*D*_ *u*(*X*, *T*_*q*_).

For example, *u*(*D*) = *u*(*D*, *T*_1_) + *u*(*D*, *T*_2_) + *u*(*D*, *T*_5_) + *u*(*D*, *T*_6_) + *u*(*D*, *T*_9_) + *u*(*D*, *T*_10_)  ( = 150 + 200 + 150 + 200 + 150 + 200)  ( = 1050). Then *u*(*BD*) = *u*(*BD*, *T*_1_) + *u*(*BD*, *T*_6_)  ( = 450 + 500)  ( = 950).

**Definition 5 .**

The transaction utility of transaction *T*_*q*_ is denoted by *tu*(*T*_*q*_), where *m* is the number of items in *T*_*q*_. Thus, *tu*(*T*_*q*_) can be defined as *tu*(*Tq*) = ∑_*j*=1_^*m*^ *u*(*i*_*j*_, *T*_*q*_).

For example, *tu*(*T*_1_) = *u*(*A*, *T*_1_) + *u*(*B*, *T*_1_) + *u*(*D*, *T*_1_)  ( = 9 + 300 + 150)  ( = 459).

**Definition 6 .**

Total utility of *D* is denoted by TU^*D*^, which can be defined as TU^*D*^ = ∑_*T*_*q*_∈*D*_ *tu*(*T*_*q*_).

For example, the transaction utilities for *T*_1_ to *T*_10_ are, respectively, calculated as *tu*(*T*_1_) = 459, *tu*(*T*_2_) = 406, *tu*(*T*_3_) = 74, *tu*(*T*_4_) = 146, *tu*(*T*_5_) = 353, *tu*(*T*_6_) = 503, *tu*(*T*_7_) = 578, *tu*(*T*_8_) = 40, *tu*(*T*_9_) = 153, and *tu*(*T*_10_) = 209. The total utility in *D* is the sum of all transaction utilities in *D*, which is calculated as (459 + 406 + 74 + 146 + 353 + 503 + 578 + 40 + 153 + 209)  ( = 2921).

**Definition 7 .**

A high-utility itemset *X* in database *D* is denoted by HUI^*D*^(*X*), which can be defined as HUI^*D*^(*X*) = ∑_*X*⊆*T*_*q*_∧*T*_*q*_∈*D*_ *u*(*X*, *T*_*q*_) ≥ *σ* × TU^*D*^.

For example, suppose a minimum utility threshold *σ* is set at 35%. An item (*D*) is considered as a HUI since its utility is *u*(*D*)  ( = 1050), which is larger than or equal to the minimum utility count as 1050 > (0.35 × 2921)  ( = 1022.35). An itemset (*BD*) is not considered as a HUI in *D* since its utility is *u*(*BD*)  ( = 950), which is smaller than the minimum utility count as (950 < 1022.35). After the above definitions, the problem statement of HUM is described below.

*Problem Statement.* Given a transactional database *D*, its total utility is defined as TU^*D*^ from *D*, a minimum utility threshold is set at 0 < *σ* ≤ 1, and the HUM is to find the complete *k*-itemsets whose utilities are larger than or equal to minimum utility count as (*σ* × TU).

Since the downward-closure property of ARM is not kept in HUM, the transaction-weighted downward closure property (TWDC) was thus proposed by two-phase model [12].

**Definition 8 .**

Thetransaction-weighted utility of an itemset *X* is the sum of all transaction utilities *tu*(*T*_*q*_) containing an itemset *X*, which is defined as TWU(*X*) = ∑_*X*⊆*T*_*q*_∧*T*_*q*_∈*D*_ *tu*(*T*_*q*_).

**Definition 9 .**

An itemset *X* is defined as a high transaction-weighted utilization itemset (HTWUI) if TWU^*D*^(*X*) ≥ *σ* × TU^*D*^.

For a 2-itemset (*AB*) in Table 1, (*AB*) is considered as a HTWUI since TWU(*AB*) = *tu*(*T*_1_) + *tu*(*T*_6_) + *tu*(*T*_7_) = (459 + 503 + 578)  ( = 1540 > 1022.35).

**Property 1 .**

The transaction-weighted downward closure (TWDC) property of two-phase model is that if an itemset *X* is a HTWUI, the subsets of *X* could be HTWUI.

Based on TWDC property of two-phase model, numerous candidates and combinational computations can be greatly reduced.

## 4. Proposed Incremental Algorithm for Transaction Insertion

In this paper, the HUI-Miner algorithm [18] is adopted to design the incremental algorithm for HUM. Before transactions are inserted into the original database, the utility-list structures are built in advance to keep not only the HTWUIs but also those itemsets which are not the HTWUIs from the original database to avoid the database rescan with transaction insertion. Since the utility-list structure is a condensed structure to keep the related information from the original database, only fewer memories are required to keep the related information of the proposed algorithm.

### 4.1. Utility-List Structure

Each entry in the utility-list structure of an itemset *X* keeps the TID numbers of *X* (TIDs), the utility of *X* in *T*_*q*_ (*Iutility*), and the remaining utility of *X* in *T*_*q*_ (*Rutility*).

**Definition 10 .**

An entry of *X* in the utility-list structure consisted of the set TIDs for *X* in of *T*_*q*_  (*X*⊆*T*_*q*_ ∈ *D*), the set of utility for *X* in *T*_*q*_ (*Iutility*), and the set of remaining utility for X in *T*_*q*_ (*Rutility*), in which* Rutility* is defined as *X*.*Rutility*(*T*_*q*_) = ∑_*i*_*j*_∈*T*_*q*_∧*i*_*j*_∉*X*_ *u*(*i*_*j*_, *T*_*q*_).

The construction procedures of utility-list structures are recursively processed for *k*-itemsets if it is necessary to process the depth-first search in the search space. The construction algorithm is then shown in Algorithm 1.

**Figure 1:**
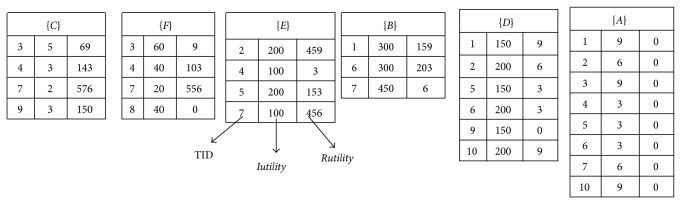
The constructed utility-list structures of 1-itemsets.

In the construction process, the itemsets are sorted in ascending order of their transaction-weighted utility (TWU). For the* Rutility* of an itemset *X* in a transaction, it keeps the rest utilities in the transaction except the processed itemset *X*. Since the TWU values of the itemsets are changed with transaction insertion, the sorted order of the utility-list structures and the* Rutility* value should also be changed. The number of inserted transactions is, however, very small compared to the original database. In the proposed algorithm, the sorted order of the itemsets in the inserted transactions follows the initially TWU ascending order of itemsets in the original database. An example to show the utility-list structures of 1-itemsets is shown in Figure 1.

**Definition 11 .**

The *X*.*Iutility*.*sum* is to sum the utilities of an itemset *X* in database *D* as

(1) $$\begin{array}{c} X.Iutility.sum=\overset{}{\underset{X\subseteq T_{q}\wedge T_{q}\in D}{\sum }}X.Iutility\left(T_{q}\right). \end{array}$$

**Definition 12 .**

The *X*.*Rutility*.*sum* is to sum the rest utilities except an itemset *X* in database *D* as

(2) $$\begin{array}{c} X.Rutility.sum=\overset{}{\underset{X\subseteq T_{q}\wedge T_{q}\in D}{\sum }}X.Rutility\left(T_{q}\right). \end{array}$$

For example, an itemset (*E*) appears in TID {2, 4, 5, 7}, and the summation of (*E*) in the database *D* is calculated as *E*.*Iutility*.*sum*( = 200 + 100 + 200 + 100)  ( = 600); the summation of rest utilities except *E* in the database *D* is calculated as (459 + 3 + 153 + 456)  ( = 1071). For more *k*-itemsets, the utility-list structures are recursively constructed until no candidates are generated for determination.

### 4.2. An Enumeration Tree

**Figure 2:**
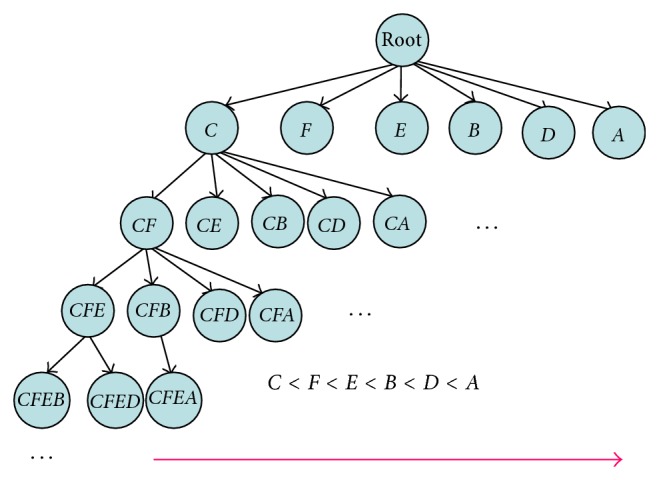
The enumeration tree.

The search space to mine HUIs is based on the enumeration tree to decide whether the supersets of the processed node *J* are required to be determined. If the summation of the* Iutility* and* Rutility* of the current processed node *J* is larger than or equal to the minimum utility count, the supersets of the processed node *J* will be generated and determined. This criterion is based on the TWDC property of the two-phase model [12]. The enumeration tree is shown in Figure 2.

**Definition 13 .**

Any extension of an itemset *X* is a combination of *X* with the itemset(s) after an itemset *X*, which is denoted by *X*′.

### 4.3. Pruning Strategy

Based on the HUI-Miner [18], a pruning strategy can also be adopted to compress the border for determination than the TWDC property.

**Property 2 .**

Given the utility-list structure of an itemset *X*, if the summation of* Iutility* and* Rutility* of an itemset *X* in *D* is less than the minimum utility count, any extension *X*′ of *X* is not a HUI.

In addition, the estimated utility cooccurrence pruning (EUCP) strategy [19] is also adopted in the proposed algorithm to further keep the relationship of 2-itemsets, thus eliminating the extension itemsets with lower utility without reconstructing the utility-list structures. The constructed EUCS is shown in Table 3.

Take the 2-itemsets (*AB*) and (*AC*) as an example to illustrate the EUCS structure. From Table 3, it can be observed that the TWU(*AB*)  ( = 1540), and TWU(*AC*)  ( = 798).

### 4.4. Proposed Incremental Algorithm

Based on the above properties inheriting from HUI-Miner and EUCS structures, the proposed incremental algorithm is described in Algorithm 2.

For the designed incremental algorithm with transaction insertion, the original database is firstly scanned to construct the utility-list structures for all 1-itemsets and the EUCS structure for each item (Lines 2–8). Similarly, the inserted transactions are also scanned to construct the utility-list structures for all 1-itemsets. Each related TWU values of items in the built EUCS are also updated by the inserted transactions (Lines 9–15). The designed merge-list algorithm is used to combine the utility-list structures from the original database and inserted transactions into an updated utility-list structures (Line 16). After that, the 1-extensions of an itemset *X* are recursively processed (Lines 17–28) by using a depth-first procedure. Each itemset *X* is then determined by the designed condition to check whether it is a HUI (Lines 18–20). If an itemset is not a HUI, its extension is then determined by the designed condition based on two-phase model (Line 21) for depth-first search. Theupdated EUCS structure is also used to prune the unpromising itemset, thus reducing the search space for mining high-utility itemsets (Lines 24–26). The construction of utility-list structure algorithm is then performed to construct the* extULs* of *X*. The proposed HUI-list-INS algorithm is then recursively performed to mine HUIs (Lines 21–29). The algorithm is then terminated until no itemsets are generated. The merge-list algorithm to combine original database and the incremental one are described in Algorithm 3.

## 5. An Illustrated Example

In this section, an example is given to illustrate the proposed incremental mining algorithm for mining HUIs with transaction insertion. Based on the TWU property, the utility-list structures for all 1-itemsets are firstly built before transactions are inserted. The inserted transactions are shown in Table 4. The original database and the profit table were, respectively, shown in Tables 1 and 2.

**Figure 3:**
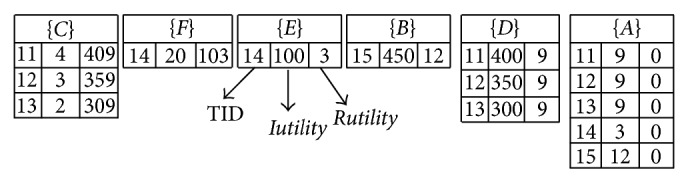
The constructed utility-list structures for the inserted transactions.

Assume the minimum high-utility threshold is also set at 35%; the updated minimum utility count for mining HUIs is calculated as (2921 + 1671) × 0.35  ( = 1607.2). First, the utility-list structures for the incremental database are also constructed for all 1-itemsets. After the construction process, the results of utility-list structures in the incremental database are shown in Figure 3.

**Figure 4:**
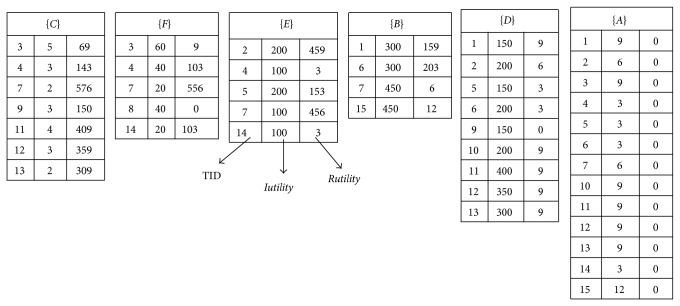
The final merged utility-list structures for the updated database.

After that, the utility-list structures from the original database and the incremental ones are merged together. For example, the utility-list structure of (*B*) in the original database is UL(*B*) = {TID, *Iutility*(*B*),  *Rutility*(*B*)} = {(1,300,159), (2,300,203), (3,450,6)}. The utility-list structure of (*B*) in the incremental database is UL(*B*)′ = {TID, *Iutility*(*B*), *Rutility*(*B*)} = {(15,450,12)}. The utility-list structures for (*B*) are then updated as {(1,300,159), (2,300,203), (3,450,6), (15,450,12)}. The other items {*A*, *C*, *D*, *E*, *F*} are processed in the same way. After that, the final updated utility-list structures are then updated and shown in Figure 4.

**Figure 5:**
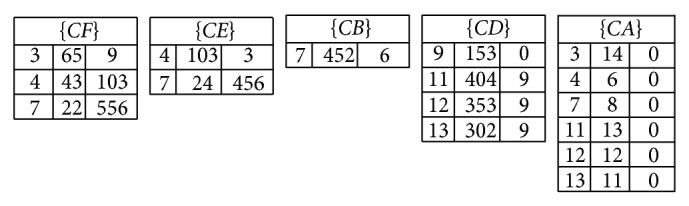
The utility-list structures for the supersets of *C*.

In this example, since the utility-list structures are sorted in ascending order of their TWU values, the item (*C*) is first processed to mine the related HUIs of (*C*). The total utility of (*C*) in the utility-list structure can be directly derived from* Iutility*, which can be calculated as (5 + 3 + 2 + 3 + 4 + 3 + 2)  ( = 22). The* Rutility* of (*C*) is calculated as (69 + 143 + 576 + 150 + 409 + 359 + 309)  ( = 2015). Since the summation of *Iutility*(*C*) is smaller than the updated minimum utility count, the summation of *Iutility*(*C*) and *Rutility*(*C*) is larger than minimum utility count as (22 + 2015 > 1607.2). Thus, the depth-search mechanism is then performed to find the supersets of the item (*C*) in the enumeration tree. The item (*C*) is then combined with item (*F*). Both of them are appeared in transactions 3, 4, and 7, which can be observed from Figure 3, to construct the utility-list structures for (*CF*). The other items (*E*, *B*, *D*, *A*) are processed in the same way. After that, the supersets of (*C*) are shown in Figure 5.

This procedure is recursively processed for all itemsets until no candidates are used to generate the utility-list structures. After all steps, the final HUIs are produced and shown in Table 5.

## 6. Experimental Evaluation

Several experiments in terms of execution time, memory consumption, and the number of patterns are conducted to show the performance of the proposed algorithm in four databases including both three real-life databases [21] and a synthetic database [22]. The two-phase algorithm [12], the state-of-the-art FHM algorithm [19], and two incremental FUP-HUI-INS [17] and PRE-HUI-INS [16] algorithms are used to evaluate the proposed algorithm. The experiments were performed in Java on an Intel Core2 Due with a 2.8 GHz processor and 4 GB main memory, running the Microsoft Windows 7 operating platform. The values of quantities and profits were assigned to the purchased items in all databases except Foodmart database. The two-phase simulation model [12] is adopted to set the quantity range from 1 to 5 and the profit range from 1 to 200 by log operation. Parameters and characteristics for four databases are, respectively, described in Tables 6 and 7.

### 6.1. Runtime

**Figure 6:**
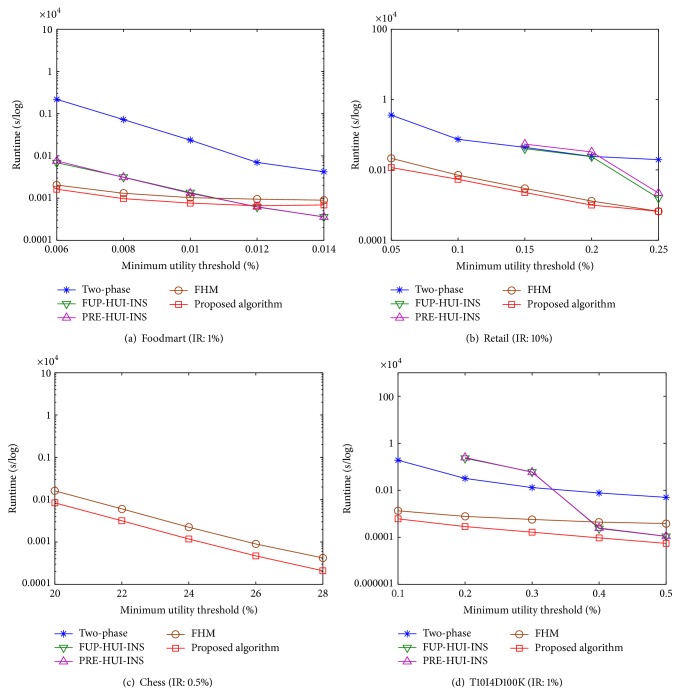
Runtime under various minimum utility thresholds.

Experiments were made to show the runtime of the proposed algorithm compared to the two-phase and FHM algorithms in batch mode and the other two incremental algorithms. The runtime includes the construction and mining phases. Experiments are then conducted to show the comparisons under various minimum utility thresholds (MUs) with a fixed insertion ratio (IR). The results are shown in Figure 6.

**Figure 7:**
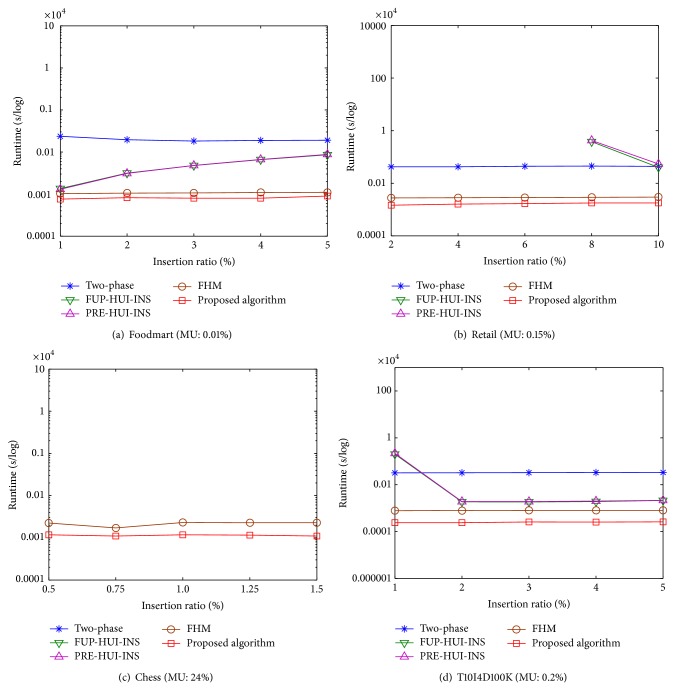
Runtime under various insertion ratios.

From Figure 6, it can be observed that the proposed algorithm has better performance than the two-phase and FHM algorithms in batch mode and the incremental FUP-HUI-INS and PRE-HUI-INS algorithms. The runtime is decreasing along with the increasing of MU. The observation is reasonable since fewer candidates of HUIs are generated when MU is set higher. When MU is set lower, the gap between the proposed algorithm and other three algorithms becomes large except the FHM algorithm, which indicates that the other three algorithms required more runtime than the proposed algorithm. Since the FHM algorithm uses the similar pruning strategies as the proposed approach, there is no great difference between them. The FHM is, however, performed in batch mode, thus requiring database rescan each time when the transactions are inserted into the original database. Experiments are then conducted to show the comparisons under different IRs with a fixed MU. The results are shown in Figure 7.

From Figure 7, it also can be observed that the proposed algorithm outperforms the other algorithms under various IRs. Take an example of Figure 7(b), the MU is set at 0.15%, and the IRs are, respectively, set from 2% to 10%, with 2% increments each time. Two incremental FUP-HUI-INS and PRE-HUI-INS algorithms have worse performance than the other algorithms. When the IR is set lower than 8%, the average runtime of two-phase algorithm is 420 seconds, the FHM is 28 seconds, and the proposed algorithm is 16 seconds. The runtime of FUP-HUI-INS and PRE-HUI-INS algorithms exceeds 10^4^ seconds. The reason is that FUP-HUI-INS and PRE-HUI-INS algorithms could have “combination explosion” problem when MU or IR is set lower. This situation may frequently occur depending on the database characteristics.

From the above results, the other algorithms have worse performance in chess database except the FHM and the proposed algorithm, which can be easily observed from Figures 6(c) and 7(c). Since the chess belongs to dense database with long patterns in the transactions, a great amount of HTWUIs are generated than those of the two-phase, FUP-HUI-INS, and PRE-HUI-INS algorithms. The FHM and the proposed algorithms apply similar pruning strategies to early reduce the unpromising itemsets, thus speeding up the computations than the other approaches.

### 6.2. Memory Consumption

**Figure 8:**
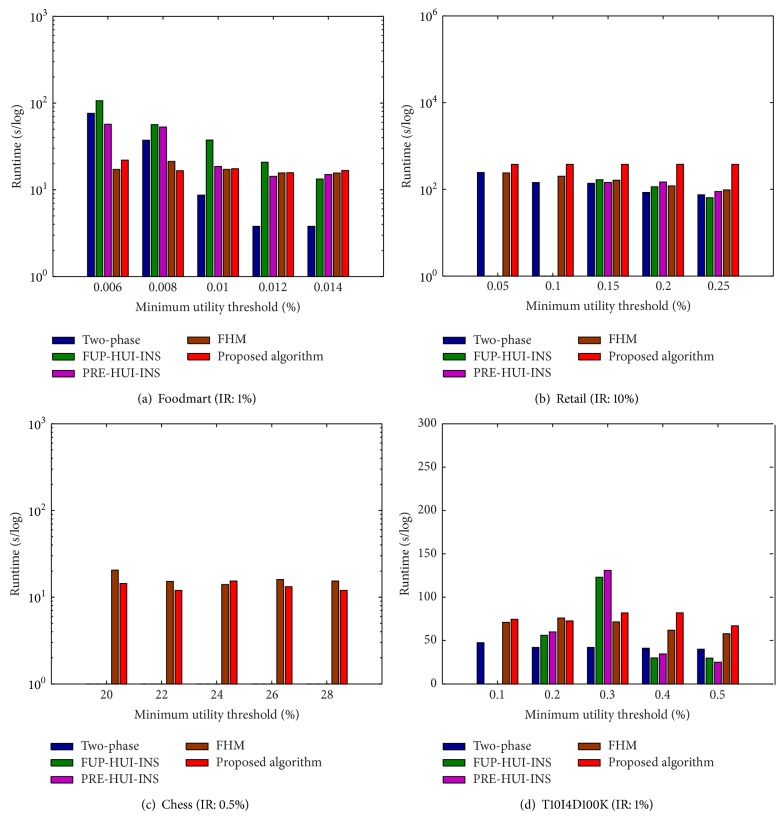
Memory consumption under various minimum utility thresholds.

Memory consumption of the propose algorithm compared to the other algorithms is then evaluated. Experiments are then conducted to show the comparisons under various MUs with a fixed IR. The results are shown in Figure 8.

**Figure 9:**
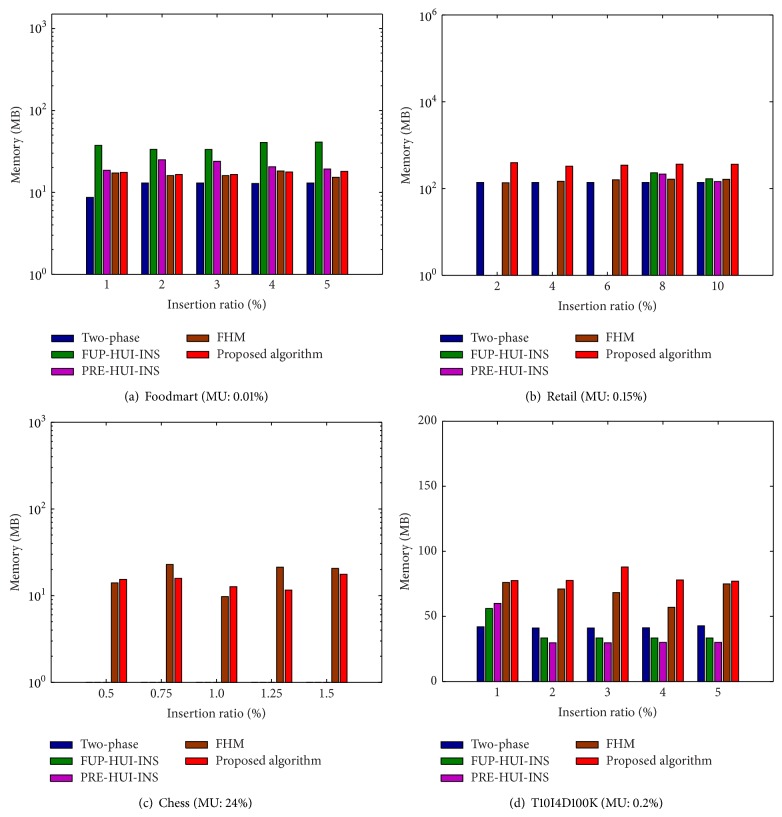
Memory consumption under various insertion ratios.

From Figure 8, it can be observed that the FHM and the proposed algorithms require steady memory along with the increasing of MUs compared to the other algorithms. This is because the fact that the FHM and the proposed algorithms are necessary to build the utility-list structures for keeping the itemsets. When MU is set lower, the proposed algorithm requires fewer memory than the other algorithms, which can be observed from Figure 8(a). Experiments are then conducted to show the comparisons under various IRs with a fixed MU. The results are shown in Figure 9.

From Figure 9(a), it can be observed that the proposed algorithm requires less memory than the other incremental algorithms along with the increasing of IRs. From Figures 9(b) and 9(d), it can be observed that the proposed algorithm requires more memory than the other algorithms. This is reasonable since more itemsets are kept in the proposed algorithm for later incremental database. Besides, the two-phase, FUP-HUI-INS, and PRE-HUI-INS algorithms cannot handle the chess database, which can be observed from Figures 8(c) and 9(c).

### 6.3. Number of Candidates and HUIs

The number of generated candidates (HTWUIs or PTWUIs) and HUIs is then evaluated to show the performance of the proposed algorithm. The two-phase and FUP-HUI-INS algorithms generate the HTWUIs. The PRE-HUI-INS generates not only the HTWUIs but also the prelarge transaction-weighted utilization itemsets (PTWUIs), and its HTWUIs is the same as the ones which are generated by two-phase and FUP-HUI-INS algorithms, so we only record the number of PTWUIs. For the FHM and the proposed algorithms, they only generate HUIs. Experiments are then conducted to show the comparisons under various MUs with a fixed IR. The results are shown in Table 8.

From Table 8, it can be observed that the two-phase, FUP-HUI-INS, and PRE-HUI-INS algorithms are performed in a level-wise approach to necessary generate the huge number of candidates for deriving the actual HUIs. Besides, the prelarge concept is adopted in the PRE-HUI-INS algorithm, thus keeping more candidates to reduce the computations of database rescan. Although the TWDC property is adopted in the two-phase mode to prune the unpromising candidate itemsets, it still requires computations to generate the amount of candidates in a level-wise way. Experiments are then conducted to show the comparisons under various IRs with a fixed MU. The results are shown in Table 9.

From Table 9, it can be observed that the number of candidates or HUIs is not dramatically increased along with the increasing of IRs. It can be concluded that different IRs would not seriously influence the number of patterns. From the observation of experiments, it can also be found that rare candidates or HUIs are generated in the incremental database. Thus, it is inefficient to rescan the original database and remine the HUIs based on the batch-mode mechanism of two-phase and FHM algorithms. The designed algorithm in real-world applications can thus be acceptable.

## 7. Conclusion

In the past, many algorithms have been proposed to efficiently mine HUIs from a static database. When some transactions are inserted into the original database, the original database is required to be rescanned to re-mine HUIs in batch mode. Fewer studies have been proposed to handle the dynamic database with transaction insertion in incremental mining. Most of them are also performed based on Apriori-like approach to generate and test HTWUIs in a level-wise way. In this paper, a novel incremental algorithm is proposed to maintain and update the built utility-list structures for mining HUIs with transaction insertion. Based on the utility-list structures, related information in the original database can thus be compressed. The proposed algorithm also applies the estimated utility cooccurrence structure (EUCS) to keep the information between 2-itemsets, thus speeding up the computations. Without the level-wise approach for generate-and-test candidates, HUIs can be easily discovered based on the designed algorithm for the incremental database. Experimental results show that the performance of the proposed algorithm outperforms that of other algorithms.

## Figures and Tables

**Algorithm 1:**
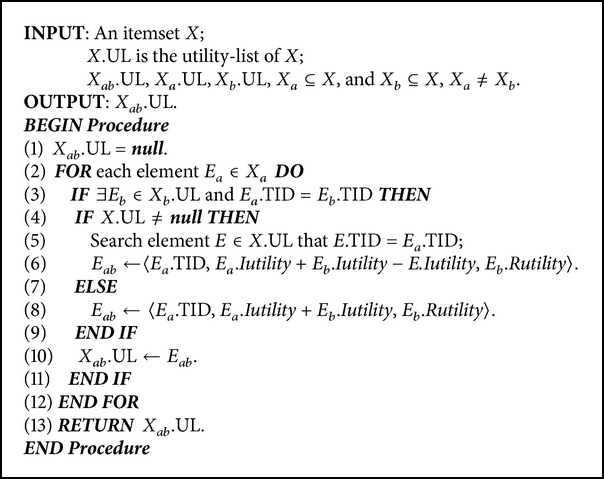
Pseudocode of utility-list structures construction algorithm.

**Algorithm 2:**
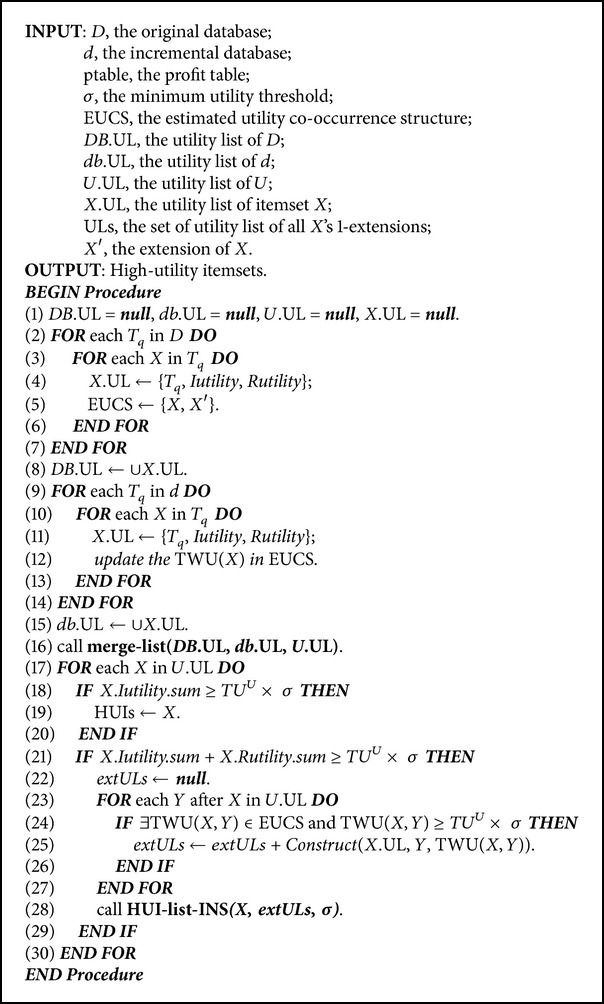
Pseudocode of the proposed HUI-list-INS algorithm.

**Algorithm 3:**
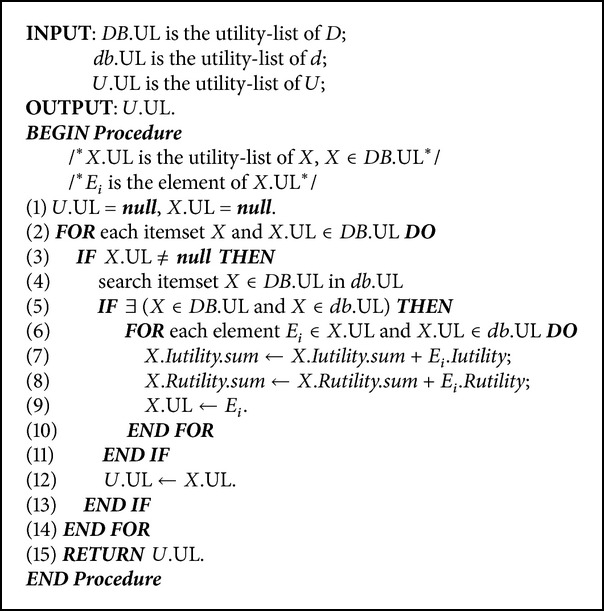
Pseudocode of merge-list function.

**Table 1:** A used example.

TID	*A*	*B*	*C*	*D*	*E*	*F*
1	3	2	0	3	0	0
2	2	0	0	4	2	0
3	3	0	5	0	0	3
4	1	0	3	0	1	2
5	1	0	0	3	2	0
6	1	2	0	4	0	0
7	2	3	2	0	1	1
8	0	0	0	0	0	2
9	0	0	3	3	0	0
10	3	0	0	4	0	0

**Table 2:** Profit table.

Item	Profit ($)
*A*	3
*B*	150
*C*	1
*D*	50
*E*	100
*F*	20

**Table 3:** Constructed EUCS structure.

Item	*A*	*B*	*C*	*D*	*E*	*F*
*B*	1540	—	—	—	—	—
*C*	798	578	—	—	—	—
*D*	1930	962	153	—	—	—
*E*	1483	578	166	759	—	—
*F*	798	578	798	0	724	—

**Table 4:** Five inserted transactions.

TID	*A*	*B*	*C*	*D*	*E*	*F*
11	3	0	4	8	0	0
12	3	0	3	7	0	0
13	3	0	2	6	0	0
14	1	0	0	0	1	1
15	4	3	0	0	0	0

**Table 5:** Final derived HUIs.

1-itemset	2-itemset
*D*: 2100	*AD*: 2007

**Table 6:** Parameter descriptions.

#|*D*|	Total number of transactions
#|*I*|	Number of distinct items
AvgLen	Average length of transactions
MaxLen	Maximal length of transactions

**Table 7:** Characteristics of used databases.

Databases	#|*D*|	#|*I*|	AvgLen	MaxLen
Foodmart	21,556	1,559	4	11
Retail	88,162	16,470	10.3	76
Chess	3,196	75	37	37
T10I4D100K	100,000	870	10.1	29

**Table 8:** Number of candidates and HUIs under various minimum utility thresholds.

Foodmart (IR: 1%)	0.006	0.008	0.010	0.012	0.014
Two-phase	399549	210788	87584	40562	22376
FUP-HUI-INS	399549	210788	87584	40562	22376
PRE-HUI-INS	3749	4490	2255	396	298
FHM/proposed algorithm	92514	25739	8240	3819	2447

Retail (IR: 10%)	0.05	0.10	0.15	0.20	0.25

Two-phase	69302	21257	11750	7641	5409
FUP-HUI-INS	—	—	11750	7641	5409
PRE-HUI-INS	—	—	765	354	212
FHM/proposed algorithm	2178	672	329	203	129

Chess (IR: 0.5%)	20	22	24	26	28

Two-phase	—	—	—	—	—
FUP-HUI-INS	—	—	—	—	—
PRE-HUI-INS	—	—	—	—	—
FHM/proposed algorithm	17370	3062	405	23	0

T10I4D100K (IR: 1%)	0.1	0.2	0.3	0.4	0.5

Two-phase	33573	16039	7526	3951	2014
FUP-HUI-INS	—	16039	7526	3951	2014
PRE-HUI-INS	—	1065	421	209	88
FHM/proposed algorithm	5576	1258	323	63	37

**Table 9:** Number of candidate itemsets and HUIs under various insertion ratios.

Foodmart (MU: 0.01%)	1	2	3	4	5
Two-phase	87584	88105	89151	90497	90757
FUP-HUI-INS	87584	88105	89151	90497	90757
PRE-HUI-INS	2255	3635	2709	2941	3219
FHM/proposed algorithm	8240	8541	9541	11030	12145

Retail (MU: 0.15%)	2	4	6	8	10

Two-phase	11673	11797	11771	11806	11750
FUP-HUI-INS	—	—	—	11806	11750
PRE-HUI-INS	—	—	—	760	765
FHM/proposed algorithm	336	333	329	330	329

Chess (MU: 24%)	0.50	0.75	1.00	1.25	1.50

Two-phase	—	—	—	—	—
FUP-HUI-INS	—	—	—	—	—
PRE-HUI-INS	—	—	—	—	—
FHM/proposed algorithm	405	426	440	455	458

T10I4D100K (MU: 0.2%)	1	2	3	4	5

Two-phase	16039	16002	15924	15996	15975
FUP-HUI-INS	16039	16002	15924	15996	15975
PRE-HUI-INS	1065	977	1032	934	899
FHM/proposed algorithm	1258	1257	1262	1263	1268
